# Structure, Oligomerization and Activity Modulation in N-Ribohydrolases

**DOI:** 10.3390/ijms23052576

**Published:** 2022-02-25

**Authors:** Massimo Degano

**Affiliations:** 1 Biocrystallography Unit, Division of Immunology, Transplantation, and Infectious Diseases, IRCCS Scientific Institute San Raffaele, via Olgettina 60, 20132 Milano, Italy; degano.massimo@hsr.it; 2 Università Vita-Salute San Raffaele, via Olgettina 58, 20132 Milano, Italy

**Keywords:** ribosides, N-ribohydrolases, structural enzymology, quaternary structure, drug design

## Abstract

Enzymes catalyzing the hydrolysis of the N-glycosidic bond in nucleosides and other ribosides (N-ribohydrolases, NHs) with diverse substrate specificities are found in all kingdoms of life. While the overall NH fold is highly conserved, limited substitutions and insertions can account for differences in substrate selection, catalytic efficiency, and distinct structural features. The NH structural module is also employed in monomeric proteins devoid of enzymatic activity with different physiological roles. The homo-oligomeric quaternary structure of active NHs parallels the different catalytic strategies used by each isozyme, while providing a buttressing effect to maintain the active site geometry and allow the conformational changes required for catalysis. The unique features of the NH catalytic strategy and structure make these proteins attractive targets for diverse therapeutic goals in different diseases.

## 1. Introduction

Ribosides are biological molecules composed of d-ribose (or a d-ribose derivative) and a nitrogenous base linked via a β-N-glycosidic bond between the C1’ atom of the pentose and a nitrogen atom from the heterocyclic aglycone. When the nitrogenous base is either adenine, guanine, cytosine, or uridine, the riboside is referred to as a nucleoside and constitutes a building block of the nucleic acid RNA. Instead, deoxynucleosides employ a reduced form of ribose, 2-deoxyribose, and the pyrimidine base thymine in place of uracil, and are selectively employed in DNA synthesis. While nucleosides are the most common ribosides in living cells, several other compounds share a similar chemical composition and structure but are employed for different purposes. For instance, the enzymatic cofactors NAD^+^ and NADPH, involved in biochemical oxidation and reduction reactions, are dinucleotides in which a phosphoester bond links adenine diphosphate to a nicotinamide riboside moiety in which the pyridine derivative nicotinamide (vitamin B3) is linked to ribose via a β-N-glycosidic bond. The free nicotinamide/nicotinic acid riboside is abundant in breast milk and also originates as a cofactor breakdown product, and together with the free base, is the preferred form of the NAD^+^ precursor for uptake by cells [1,2,3]. Nucleosides in tRNA molecules often undergo post-transcriptional modification, and when these molecules are degraded, their breakdown products are ribosides with modified purine or pyrimidine bases [4]. In plants, the class of cytokinin hormones includes adenine base derivatives that are synthesized in the roots and delivered to target districts through circulation in the xylem in the form of ribosides, which are later reprocessed to the active hormone [5]. Ribosides may also display a modified ribosyl moiety, a prominent example being the thioribosides S-adenosylmethionine (SAM) or 5’-methylthioadenosine (MTA), where a sulfur atom rather than an oxygen is covalently linked at position C5’. SAM is a ubiquitous methyl donor in biochemical reactions, and MTA is part of the methionine cycle for polyamine synthesis [6]. Hence, ribosides include a vast landscape of diverse molecules, not limited to nucleic acid components, and are involved in many different biochemical and physiological processes.

The free ribosides are mostly intermediate metabolites. Indeed, ribosides can cross the cell membrane, taking advantage of equilibrative nucleoside transporters, since they lack the negative charges localized on the phosphate groups present in ribotides, including nucleotides [7,8]. Thus, this property of ribosides allows their use as a carrier of nitrogenous bases in the circulation and across cell membranes. In the human digestive system, nucleic acids introduced in the diet are hydrolyzed to oligonucleotides by nucleases, followed by the action of phosphodiesterase enzymes to yield nucleotides, and ultimately, dephosphorylation by nucleotidases to allow the nucleosides to enter the intestinal villi. Once inside cells, the fate of ribosides can be substantially dual, undergoing either phosphorylation to the corresponding ribotide monophosphate in a reaction catalyzed by specific kinases, or cleavage of the N-glycosidic bond to release the nitrogenous base. The detachment of the nitrogenous base from the ribosyl moiety is preferred in most cells as it allows for high flexibility in recycling the sugar and base components according to the immediate needs. The ribose moiety can be diverted either to enter biosynthetic processes, such as nucleotide or amino acid synthesis, or for energetic metabolism via the pentose phosphate pathway to provide glycolytic intermediates. The aglycone can undergo two opposite fates, (a) the condensation with 5-phosphoribosyl-α-1-pyrophosphate (PRPP) to yield the corresponding mononucleotide/ribotide, or (b) base degradation, for instance, via an oxidative pathway for purines or a reductive one for pyrimidines. Free ribosides can also be endowed with biological activity. In mammals, extracellular adenosine performs an important role in the regulation of the sleep/wake cycle, binding to specific purinergic receptors [9]. Cytokinin ribosides [10] not only act as base transporters but are also involved in the response to abiotic stress in plants.

Although most organisms can synthesize the nitrogenous bases that are components of nucleosides and other ribosides, the adequate activity of the aglycone salvage pathways is absolutely central to normal cellular life. For instance, the seminal role of nucleobase recycling in mammals is highlighted by the existence of human immunodeficiencies linked to inefficient N-glycosidic bond cleavage in nucleosides, such as purine nucleoside phosphorylase deficiency, or PNP-SCID. The reduced nucleoside phosphorylase activity limits the availability of nitrogenous bases for nucleic acid synthesis and leads to a limited proliferative capacity of T cells [11]. Detachment of the base from the thioribosyl moiety of SAM and MTA is crucial for the maintenance of an adequate balance between intersecting pathways, such as the methionine cycle and polyamine biosynthesis. Clearly, organisms that are auxotrophic for specific nitrogenous bases rely even more on the activity of the appropriate lytic enzymes. The purine-auxotrophic protozoan parasites of the *Trypanosoma* genus require the activity of N-ribosidases to utilize the nucleobases taken up from the hosts [12], while *Trichomonas vaginalis* is unable to synthesize both purine and pyrimidine bases de novo [13]. N-glycosidic bond cleavage is also required in yeast for the metabolism of pyridine nucleosides that harbor the nicotinamide/nicotinic acid moiety of the redox cofactors NAD^+^ and NADPH [14]. It is thus clear how organisms are faced with the necessity of employing highly efficient and selective enzymes in the N-glycosidic bond cleavage process or ribosides, paralleling the structural and functional diversity of the substrates.

Two principal enzymes involved in N-glycosidic bond cleavage have been characterized extensively, the nucleoside phosphorylases (NPs) that perform a phosphorolysis of the ribose-base bond, and the nucleoside hydrolases (NHs) that employ a water nucleophilic attack to detach the base from ribosides. Here, I review the existing information on their roles, mechanisms, structure-activity relationship in NHs, and how the NH fold can be employed for different biological processes. The natural homo-oligomerization to distinct quaternary structures parallels the enzymatic properties and substrate specificities of NHs. The absence of NH-encoding genes in mammals makes these proteins attractive targets for diverse therapeutic approaches against different diseases, from chemotherapeutic treatment of parasitic infections to vaccines or activation of prodrugs following gene delivery to tumoral cells.

## 2. Review

### 2.1. Enzymatic N-Glycosidic Bond Cleavage in Biological Systems

**Figure 1:**
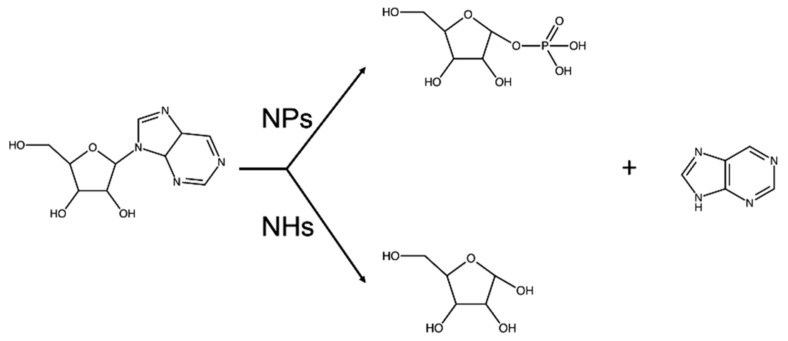
N-ribosidic bond cleavage in biological systems. Phosphorolysis catalyzed by NPs or hydrolysis by NHs of ribosides (here, purine riboside is shown as a general example) releases the nitrogenous base, and either ribose 1-phosphate or ribose is formed.

N-glycosidic bond cleavage in ribosides takes place in several biochemical pathways. Despite the existing diversity in the chemical nature of the nitrogenous base and ribosyl moiety modification, a general scheme of the destiny of the cleavage product can be described (Figure 1). The cleavage of the covalent link between the pentose and aglycone takes place via two primary reactions, phosphorolysis or hydrolysis.

#### 2.1.1. N-Glycosidic Bond Phosphorolysis

A common strategy in most organisms is the cleavage of the N-glycosidic bond in nucleoside substrates via a phosphorolytic reaction catalyzed by a family of nucleoside phosphorylases (NPs) [15,16]. All NP enzymes bind an inorganic phosphate ion to the active site, directing its nucleophilic attack to the C1’ atom of the nucleoside substrate and yielding the free base and the ribosyl moiety linked to the negatively charged phosphate [17] that is thus retained in the cell cytosol. For ribonucleosides, the ribose 1-phosphate product can be isomerized to ribose 5-phosphate and either converted to PRPP for nucleotide synthesis or entered into energetic metabolism via the pentose phosphate pathway. The free nitrogenous base obtained after phosphorolysis can react with PRPP in a phosphoribosyltransferase-(PRTase) catalyzed reaction to yield the corresponding nucleotide monophosphate. The NP enzymes differ in their quite strict substrate specificity, preferring purine, pyrimidine, or thionucleosides as active-site ligands. Indeed, purine-specific (PNP) [15], pyrimidine-specific (PyNP) [18], and MTA-specific (MTAP) [6] enzymes have been described and extensively studied. NPs generally show weak hydrolytic activity, the relevance of which in the cellular context at physiological nucleoside and phosphate concentrations is unclear.

#### 2.1.2. N-Glycosidic Bond Hydrolysis

An alternative strategy to phosphorolysis is the reaction of the riboside with water and obtaining the release of the nitrogenous base through a hydrolytic reaction catalyzed by N-ribohydrolases (EC 3.2.2, including ECs 3.2.2.1, 3.2.2.7 in the BRENDA classification, and the literature also referred to nucleosidases, NHs). Yet, the activity of N-ribohydrolytic enzymes is redundant in the process of nucleobase salvage, paralleled by the efficient phosphorolysis catalyzed by NPs. Moreover, the NH-catalyzed reaction releases ribose as a product, which requires ATP-dependent phosphorylation via ribokinases to enter the catabolic and anabolic pathways [19]. The current view is that NH activity is linked to highly specialized functions in each organism, and its physiological role can extend beyond the salvage of nucleobases to, among others, the uptake of other compounds, such as pyridines, or the activation of cytokinin hormones in plants. NH activity is notably absent in mammals, in whom NH-encoding genes were apparently lost during evolution.

### 2.2. Biological Functions of Nucleoside/Riboside Hydrolases (NHs)

NH enzymes were originally envisioned as an alternative to NPs for the rescue of nucleobases from nucleosides. Their involvement in other pathways has become apparent only in recent years, and it is becoming increasingly clear that NHs have diversified roles in different organisms.

#### 2.2.1. NHs from Protozoa

Protozoan parasites of the *Trypanosoma* genus are purine auxotrophic [20] and rely on the bases obtained from the host for the synthesis of the corresponding nucleotides [12]. To this end, purine nucleosides are imported into the parasite via neutral nucleoside transporters [8,21], where they either undergo direct phosphorylation via nucleoside kinases or N-glycosidic bond cleavage. Unlike other organisms, notably the mammalian hosts, trypanosomes do not display purine-specific NP activity. Instead, the purine base is primarily (>82%) obtained through a hydrolytic reaction catalyzed by NHs [22]. NH activity has been demonstrated in many trypanosomatids, and the responsible enzymes have been purified from the protozoan cells [22,23,24,25]. Among the first NHs purified to homogeneity and extensively characterized at the enzymatic level was the non-specific, inosine-uridine-preferring isozyme (termed IU-NH, for inosine-uridine-preferring nucleoside hydrolase) from *Crithidia fasciculata* [24]. The catalytic efficiency of the enzyme (k_cat_/K_M_) ranges from 4.5·10^3^ for cytidine to 7.6·10^4^ M^−1^·s^−1^ for inosine, and the K_M_ values are between 380 and 4700 µM, at least threefold greater for pyrimidine nucleosides. Deoxynucleosides, lacking either the 2’, 3’, or 5’ hydroxyl, are substrates with very low turnover, highlighting for the first time the strict ribose specificity of this NH that is a common feature of all isozymes so far characterized. The transition state structure of the enzymatic reaction shows a marked oxonium ion character, with a partial double-bond character between the C1’ and O4’ atoms, an elongated N-glycosidic bond, and an incoming enzyme-directed water molecule performing the nucleophilic attack. Distortion of the ribosyl conformation was highlighted by a kinetic isotope effect at the C5’-linked hydroxymethyl group [26,27]. The involvement of two ionizable residues in catalysis but not substrate binding suggested an acid-base catalytic mechanism, and site-directed mutagenesis showed that the residue His241 is involved in the proton-transfer process to the nitrogenous base leaving group. The catalytic power of the enzyme is largely due to ground-state destabilization through geometric distortion of the substrate to a high-energy conformation rather than leaving-group activation via protonation of the nitrogenous base. Indeed, the high catalytic activity on the synthetic substrate 4-nitrophenyl riboside (pNPR), the hydrolysis of which does not require leaving-group protonation, showed that out of -17.7 kcal/mol, representing the decrease in activation energy for the enzyme-catalyzed reaction, -13.3 kcal/mol derived from ribosyl distortion to the oxonium ion geometry [28]. This mechanism is apparently common to all NHs belonging to the structural group I (see below).

This mechanism, however, is not the only biochemical strategy that can be employed to perform the hydrolysis of the ribose-base bond. The enzymatic properties of the inosine-guanosine-preferring NH from *C. fasciculata* [22] showed that a single protonated residue is required for inosine hydrolysis. Enzymatic studies on the purine-specific NH from *Trypanosoma vivax*, a pathogen causing nagana disease in cattle and wildlife, showed much lower K_M_ values for the purine nucleoside substrates, consistent with the necessity of high efficiency at low substrate concentrations [29]. Moreover, the reaction mechanism activates the leaving group through both aromatic stacking of an active site Trp residue and an intramolecular hydrogen bond between the ribosyl O5’ with the substrate purine ring to raise the pK_A_ of the N7 nitrogen and allow protonation from the solvent. An enzyme-directed water molecule is responsible for the nucleophilic attack on the C1’ atom of the substrate [30]. Indeed, the catalytic power of the enzyme relies less on ribosyl distortion compared to the IU-NH from *C. fasciculata*. The isozyme from *Trypanosoma brucei brucei*, also a parasite of non-human vertebrates but closely related to the human pathogens *T. b. gambiense* and *T. b. rhodesiense* causing sleeping sickness, confirmed similar kinetic properties [31], thus clearly showing that NH enzymes can segregate into subgroups with quite distinct enzymatic properties that are likely due to distinct arrangement of amino acid residues involved in catalysis.

#### 2.2.2. Bacterial and Archaeal NHs

**Figure 2:**
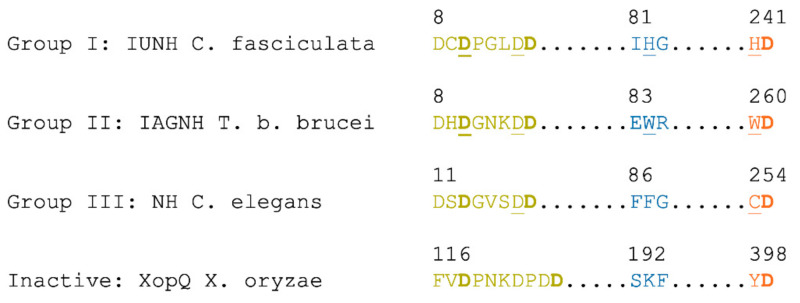
NH fingerprint sequence in NH enzymes and NH-like proteins. In bold are the Asp residues that are involved in the coordination of the Ca^2+^ ion. Underlined are the amino acid residues in each group that have been demonstrated through site-directed mutagenesis to be involved in catalysis. The color scheme matches the one used in the active site representations. The sequences are taken from the deposited PDB files (PDB codes 2MAS, 4I71, 5MJ7, 4P5F).

The presence of NHs in bacteria is a relatively recent discovery that followed the identification of the NH-fingerprint sequon [28,32], allowing the annotation of ORFs and putative proteins in genomic sequences (Figure 2). Although the well-established activity of NPs in nucleobase salvage in bacteria makes the NH activity redundant, bacterial genomes encode for varying numbers of NH-like genes, at least one being systematically present [33]. The *Escherichia coli* genes *ybeK*, *yeiK,* and *yaaF* encode three functional NHs, two (RihA/YbeK and RihB/YeiK) with a preference for pyrimidine nucleosides (CU-NH) [33,34] and one (RihC/YaaF) hydrolyzing both purine and pyrimidine nucleosides (IU-NH) [35]. Deletion of the three genes in *E. coli* did not result in apparent phenotypes or growth defects, at least under the growth conditions tested [36]. In the spore-forming bacilli, such as *Bacillus cereus* and *Bacillus anthracis*, a purine-specific NH is found in the exosporium. The NH from bacilli is involved in the degradation of purine nucleosides to prevent inosine- and adenosine-induced sporulation [37,38,39]. The *Pseudomonas aeruginosa* NH participates in the synthesis of autoinducer-2, and it has been postulated to act through the recycling of MTA [40,41,42]. However, no reports on the characterization of the enzyme and its specificity are available thus far. Instead, NH activity on lumenal uridine is a fingerprint of pathogenic gut flora in *Drosophila*, both favoring uracil secretion and activating through a free ribose the transcription of genes involved in acyl homoserine lactone synthesis, thus playing a key role in host-flora interaction [43]. An NH-like gene (*iunH*) is present in the *Mycobacterium tuberculosis* genome, but mycobacteria engineered to prevent *iunH* gene expression are viable and infectious, thus indicating that the NH enzyme is not involved in key steps of the pathogen’s life cycle [44,45]. The archaeon *Sulfolobus solfataricus* also bears two NH-like genes, encoding for one purine-specific and one pyrimidine-specific enzyme endowed with very high thermal stability [46,47]. A growing number of studies are demonstrating how the NH structural scaffold can be used for other purposes than riboside hydrolysis. Members of the plant bacterial pathogens *Xanthomonas* spp. secrete an NH-like protein that differs from the “classic” NH enzymes by including an 80-amino-acid amino terminal addition [48,49,50]. Members of this group are referred to as XopQ (*Xanthomonas* outer protein Q) or HopQ and promote bacterial virulence. XopQ from *X. oryzae* is devoid of the nucleosidase catalytic activity, but binds to ADP-ribose at the site that is structurally equivalent to the catalytic site of NHs [51]. While the precise mechanism mediating XopQ virulence is unknown, its recognition by the ROQ1 resistosome plant complex leads to activation of the immune response and resistance to pathogen invasion [52]. NH-like domains have been identified in multidomain proteins from bacteria such as *Saccharophagus degradans*. The NH domain in the putative protein Sde_0182 follows three carbohydrate-binding domains and a beta-sandwich domain and is inactive toward purine and pyrimidine nucleosides. Thus, the current hypothesis is that the NH-like domain in this protein is employed as a furanose sugar-interacting module [53].

#### 2.2.3. NHs in Plants

The purine bases can become a source of nitrogen under starvation conditions in plants and thus play an additional, essential role in their physiology. Adenosine-specific and non-specific NH enzymes have been characterized from yellow lupin [54] and Alaska pea seeds [55], respectively. Several putative NH genes have been annotated based on the presence of the amino-terminal NH-fingerprint sequence, and the products of the NH1 and NH2 genes from *Arabidopsis thaliana* have also shown activity toward the common nucleosides, including inosine, adenosine, and xanthosine, which can become a nitrogen source [56]. Interestingly, the two proteins show a unique mutual activation through the formation of a heterocomplex that enhances and modulates the catalytic efficiency of the homomeric enzymes [57]. The physiological role of NHs that act both at the intracellular and extracellular levels extends beyond nucleotide metabolism. For instance, a decrease in NH activity encoded by the NHR1 enzyme causes a delay in bud formation in *Physcomitrella patens*, thus suggesting an involvement in the metabolism of plant hormones such as cytokinins [58]. Moreover, NHR1 is crucial for plant growth when adenosine is provided as the sole nitrogen source. Analysis of the nucleoside, nucleobase, and cytokinin amounts in *P. patens* NH mutants confirmed significant alterations of the purine and hormone metabolism.

#### 2.2.4. NHs in Other Eukaryotes

NHs have been identified in yeast (*Saccharomyces cerevisiae*) [59], nematodes (*Caernorhabditis elegans*) [60], diptera (*Aedes aegypti*) [61], and amphibians (*Xenopous laevis*) [33]. The NH from the mosquito *A. aegypti*, the vector of dengue virus, is injected into the host during the bloodmeal and degrades circulating adenosine to prevent mast-cell activation via activation of purinergic receptors, effectively working as a local anesthetic. The *C. elegans* NH has broad substrate specificity, hydrolyzing both purine and pyrimidine nucleotides, but the physiological role of the enzyme is unknown. The most compelling functional data on eukaryotic NHs are on the yeast URH1 (Urh1p) enzyme, an NH with a strong preference for uridine as a substrate. Initially characterized in the 1970s [62,63], the enzyme was shown to participate in the metabolism of the pyridine nucleoside nicotinamide riboside, a NAD^+^ precursor. URH1 activity critically modulated the activation of sirtuins, which are central players in extending the lifespan of yeast cells [14]. Hence, the modulation of the substrate preference of the NHs allows their employment in different physiological settings. Why the NH genes were lost during evolution to mammals remains a thought-provoking, yet not addressed, question.

### 2.3. NH Tertiary Structure

NH enzymes (and related proteins) have a unique fold that is classified as an α/β three-layered sandwich [32]. Within NH proteins so far characterized, sufficient diversity (Table S1) is observed to allow the definition of three structural groups that parallel distinct substrate specificities and quaternary structures [33], and a distinct set of catalytically-inactive NH proteins. The NH groups are characterized by both differences in the content of secondary structure elements and catalytic residues, specifically one amino acid involved in leaving-group stabilization [33]. Most NH proteins whose structure has been experimentally determined belong to group I, including the non-specific and pyrimidine-specific isozymes. Phylogenetic analysis of their amino acid sequences (Figure S1) shows how the different structural groups segregate into distinct tree branches. Here, I discuss the properties, similarities, and differences between members of the structural groups.

#### 2.3.1. NH Structural Group I

**Figure 3:**
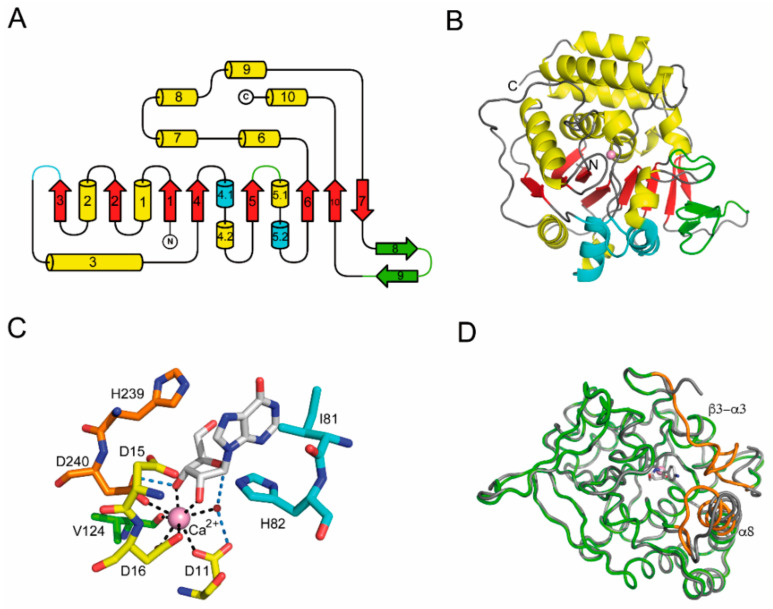
Group I NH structure. (**A**) Overall topology diagram of group I NHs. The major secondary structural elements shared by all group I NHs so far characterized are indicated (α-helices as cylinders, β-strands as arrows). Occasionally, additional short helical segments are identified through automated analysis, thus the numbering may differ. Strands β8 and β9 (green) are components of the major interaction surface between group I monomers for tetramer assembly, while elements colored in cyan compose the minor interaction surface. (**B**) Ribbon diagram of the monomer of the epitomic *C. fasciculata* IU-NH (PDB code 2MAS). (**C**) Active site architecture of group I NHs. Here, the complex between the E. coli RihB/YeiK enzyme and inosine is shown. The Ca^2+^ ion octacoordination is composed of three Asp residues from the NH sequon, the carbonyl oxygen of a non-conserved amino acid (commonly Thr, Val or Ile), one water molecule, and the O2’ and O3’ ribosyl hydroxyls. In the absence of a substrate, two ordered waters occupy the hydroxyl positions. Hydrogen bonds between the enzyme, substrate, and catalytic water are colored blue. Amino acids are colored according to the scheme in Figure 2. (**D**) Conformational changes in group I NHs on binding of active site ligands. The α3-β3 loop and helix α8 are highly flexible in the structure determined in the absence of a ligand (gray tube), and undergo a transition to a defined conformation when active site ligands are bound (green tube, with the segments undergoing a structural transition colored orange).

The first NH crystal structure determined was of the inosine-uridine-preferring, but substantially non-specific, isozyme from the trypanosomatid parasite *C. fasciculata* [32]. Highly similar structures have since been identified in bacterial, archaeal, and plant NHs with either pyrimidine nucleoside specificity or a broad tolerance for both purine and pyrimidines in the aglycone (Table S2). The polypeptide folds into a single domain, composed of a central eight-stranded mixed β-sheet surrounded by α-helices. The first six β-strands are parallel to each other, each one followed by one α-helix to constitute a series of right-handed β-α-β motifs that closely resemble the Rossmann fold. The third α-helix is part of an extended “crossover” junction segment, leading to strand β4 that is parallel to β1 and to the topological switch point that hosts the substrate binding site. After strand β6, the polypeptide folds into three separate α-helices that stack toward one side of the core β-sheet, and together with the C-terminal α10 helix, complete the enzyme active site. Two additional β-strands, one parallel and one antiparallel, complete the central β-sheet. The polypeptide arrangement also includes two additional short β-strands, antiparallel to each other and not interacting with the core structure (Figure 3).

In all group I NHs, the enzyme active site is in a cavity at the C-terminal end of strands β1 and β4, with contributions from several loop regions and residues from helices α8 and α9. The bottom of the cavity is characterized by three Asp residues, two part of a conserved DxDxxxDD fingerprint sequence in the β1-α1 region and one from approximately 230 amino acids downstream (Figure 2). These negatively charged residues coordinate a Ca^2+^ ion, the octacoordination of which is completed in the unliganded NHs by the carbonyl oxygen of a Thr/Ile residue and three ordered water molecules. Binding of nucleoside substrates or transition state-like inhibitors leads to the displacement of two water molecules by the O2’ and O3’ ribosyl hydroxyls (Figure 3). The remaining water molecule is poised for the nucleophilic attack in the hydrolytic reaction from the opposite side of the β-N-glycosidic bond, yielding α-ribose as the reaction product that undergoes mutarotation on release from the enzyme. Both the α and β anomers of ribose can bind to the NHs active sites, as seen in the *E. coli* RihA structure. Other ligands such as glycerol, a common cryoprotectant in X-ray cryocrystallography experiments, can mimic part of the ribosyl moiety and are typically observed in NH active sites [33].

The activation of the leaving group takes place through enzyme-directed proton transfer, as demonstrated from the enzymatic activity profiles at different pH values [24]. An involvement of the conserved His residue immediately preceding the last Asp residue in the Ca^2+^ coordination sphere (Figure 2) has been demonstrated through site-directed mutagenesis for both the non-specific IU-NH from *C. fasciculata* and the *E. coli* RihB/YeiK pyrimidine nucleosidase. Molecular dynamics simulations suggest that the residue is part of a protein relay network involving other amino acids, including the third Asp residue of the fingerprint sequence [64].

Regardless of their origin, all group I NHs hydrolyze pyrimidine nucleosides, albeit with different catalytic efficiencies. Instead, purine nucleosides require the presence of two hydroxylated residues in the α8 helix for efficient hydrolysis. Specifically, two Tyr residues extending in the active site are apparently necessary to form a proton-relaying network to achieve N7 protonaton. Indeed, the introduction of two Tyr residues at positions 223 and 227 in the corresponding helix of the *E. coli* pyrimidine-specific NH RihB/YeiK was sufficient to cause a 50-fold increase in k_cat_/K_M_ for the substrate inosine [65].

In the unliganded NHs, two highly flexible regions are present. Indeed, a) the initial portion of the crossover loop between the secondary structure elements β3 and α3, and b) the C-terminal region of helix α8, are usually not resolved in the electron-density maps of enzymes in the absence of active-site ligands. These regions become ordered when the enzyme binds a ligand mimicking the nitrogenous base, as was shown for both the IU-NH from *C. fasciculata* bound to an iminoribitol-based compound [66] and the *E. coli* CU-NH RihB/YeiK bound to the slowly hydrolyzed inosine substrate [65] (Figure 3). The β3-α3 junctional loop becomes ordered, providing both hydrophobic and polar contacts with the base aromatic ring via a conserved Val/Ile-His dipeptide part of a single helical turn. The α8 helix undergoes a rotation about its axis and a swiveling movement to bring its residues near the substrate, establishing hydrogen bonds to the purine or pyrimidine ring substituents. This combined conformational change, studied through steered molecular dynamics for the *E. coli* RihB/YeiK, may represent a rate-limiting step for nucleoside hydrolysis and is influenced by the protonation of the conserved His residue in the β3-α3 loop [67].

#### 2.3.2. NH Structural Group II

**Figure 4:**
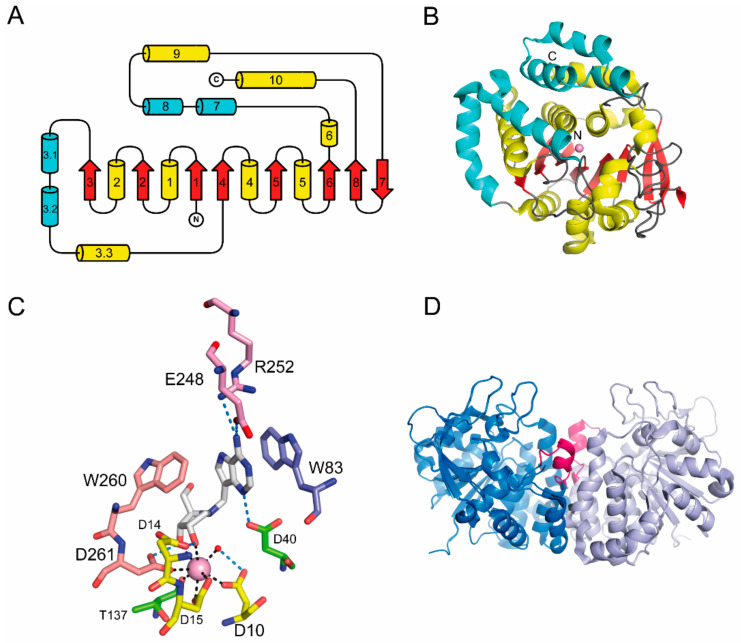
Group II NH structure. (**A**) Topology diagram of group II NHs. Four helical segments (α3.1, α3.1, α7, and α8) are part of the monomer-monomer interface. (**B**) Ribbon diagram of the monomer of the *T. brucei brucei* IAG-NH (PDB code 4I71). (**C**) Active site of group II NHs. The *T. brucei brucei* IAG-NH in complex with the UAMC-0063 inhibitor shows the aromatic stacking between the aglycone and two signature Trp residues, along with specific hydrogen bonding to the base substituents. (**D**) Flexible loop conformational selection. The flexible loop in unliganded IAG-NH spanning residues 245–255 becomes ordered (pink) on binding of substrates or competitive inhibitors.

Trypanosomal NHs include enzymes that are truly purine nucleoside-specific (IAG-NHs) and are characterized by K_M_ values in the low micromolar range, consistent with the requirement for high catalytic efficiency at low substrate concentrations for nucleobase uptake [23,29]. The IAG-NHs from *T. vivax* and *T. brucei brucei* share the overall NH fold of the group I proteins (Figure 4 and Table S2), but display distinct structural features that explain the differences in substrate specificity, catalytic mechanism, and quaternary structure. The most striking differences reside in the crossover segment linking strands β3 and β4, which in group II NHs contains two additional α-helices. These helices, as will be discussed later, are central in group II NH dimerization.

While the Ca^2+^ ion and its coordinating residues are conserved as in the group I NHs, the portion of the active site interacting with the purine ring in group II is remarkably different. Two Trp residues substitute the two His present in the β3-α3 loop and immediately before the last Ca^2+^-coordinating Asp in the sequence. The indole rings of these amino acids provide aromatic stacking interactions with the purine ring, resulting in lower K_M_ values for purine nucleosides compared to the group I NHs [68]. Secondly, the stacking between the π systems of the substrate and the enzyme leads to an increase in the pK_A_ of the N7 atom, allowing protonation of the leaving group from the solvent (Figure 4). Specifically, the Trp83Ala substitution in the β3-α3 loop, as studied in the *T. vivax* isozyme, increases the K_M_ value by a factor of three, while the Trp260Ala mutation reduces by three orders of magnitude the catalytic rate [30,69]. Thus, the aromatic residues in the group II NHs provide a unique framework for substrate selection and an elegant alternative to direct enzyme-mediated protonation as a means of activation of the leaving group.

Group II NHs also undergo a flexible-to-tight structural transition since the loop connecting residues 245–255 becomes visible on electron-density maps on the binding of substrates or active-site inhibitors (Figure 4). The dynamics of the loop movement were first shown for the *T. vivax* enzyme [70] and later confirmed, too, for the *T. b. brucei* homolog, and are apparent in the structure of the enzyme co-crystallized with the transition state-like inhibitor immucillin-H. Instead, the compound soaked in the pre-formed crystals does not undergo the loop-locking transition, underscoring how crystal contacts may hamper the full appreciation of structural changes on ligand binding. The structural reorganization of group II NHs achieves an effective shielding of the reaction center from the bulk solvent and allows a stereospecific nucleophilic attack by a Ca^2+^-coordinating, enzyme-directed water molecule.

Purine-specific NHs are not limited to parasites of the *Trypanosoma* and *Leishmania* genera but are also found in bacilli [37] and insects [61], thus showing how the catalytic activity of group II NHs can be taken advantage of in different organisms. Again, this NH activity parallels that of the PNPs that are expressed in the vast majority of living cells; thus, it is also employed in different biochemical pathways such as adenosine degradation in the host during blood meals by insects, or purine nucleoside degradation to induce sporulation in bacilli.

#### 2.3.3. NH Structural Group III

Based on the nature of the active-site residues that are present in the β3-α3 loop (His vs. Trp in group I and II NHs) and before the last Asp in the Ca^2+^ coordination sphere (again a His in group I vs. a Trp in group II), a third structural group was anticipated. Indeed, a group of NH proteins from metazoa including *C. elegans* and *X. laevis* display a Cys residue rather than Trp260 or His241 [33,71]. Structural characterization of the *C. elegans* NH showed that it shares the highest catalytic similarity to group II NHs, but displays an overall structure that is more similar to group I NHs, including its tetrameric quaternary structure [71]. Site-directed mutagenesis demonstrated that the Cys residue is involved in the leaving-group activation, albeit through a yet-unidentified mechanism. More structures of homologs harboring the catalytic Cys and their complexes with substrates are necessary to fully understand the enzymatic mechanism in this NH subgroup. The *C. elegans* nematode is a convenient model for genetic analysis and may represent an interesting model to understand the physiological role of NHs in higher eukaryotes.

#### 2.3.4. NH Fold in Catalytically-Inactive Proteins

The *Xanthomona oryzae* XopQ virulence factor contains a domain sharing the overall NH fold, but amino acid insertions and deletions compared to both the group I and group II NHs result in a much larger active-site volume [50,51]. Variations in the residues that mediate oligomerization in both group I and group II NHs are also apparent. As a result, XopQ is present in a monomeric form in solution. XopQ retains the Ca^2+^-binding site, but no residues to activate the leaving-group nitrogenous base, and is inactive towards the common nucleosides. The exact reaction catalyzed by XopQ, if any, remains elusive. Interestingly, the larger binding site of XopQ allows the binding of ADP-ribose via the ribosyl moiety of the latter, coordinating the binding site Ca^2+^ ion as RNA nucleosides do in active NHs. Binding of ADP-ribose through the ribose hydroxyls induces a large conformational change in the protein through the hinged movement of helix α12 that leads to the “wrapping” of the protein around the bound dinucleotide. Interestingly, the protein was co-crystallized in the presence of cyclic ADP-ribose, but ADP-ribose was instead found in the crystals [51]. Since the crystallized solution did not promote non-enzymatic hydrolysis of cyclic ADP-ribose, the hypothesis was put forth that XopQ may possess such enzymatic activity. The physiological relevance of this finding remains to be assessed, although it is intriguing that the physiological interaction of XopQ is with a nucleotide-binding leucine-rich receptor (NLR) that is endowed with NAD^+^ nucleosidase activity and the reaction product of which is indeed ADP-ribose [52].

#### 2.3.5. “Non-Standard” NH Enzymes

Both *C. fasciculata* [22] and *T. brucei brucei* [72] encode for an NH isozyme with a marked specificity for inosine and guanosine (IG-NH), while adenosine is a poor substrate with a low turnover number. The overall fold of the *T. brucei brucei* IG-NH is more similar to group I NHs, with whom the isozyme also shares a tetrameric quaternary structure. However, the active site contains two Trp rather than His residues, thus likely activating the leaving-group purine via aromatic stacking, as is the case for group II purine-specific NHs. Hence, this IG-NH represents a “hybrid” structure, sharing properties of both group I and II NHs.

The IAG-NH and CU-NH from the archaeon *S. solfataricus* also display peculiarities that make them exceptions to the principles inferred from the other NHs. The *S. solfataricus* IAG-NH is unusual in its kinetic parameters, in particular, K_M_ values that are 4- to 60-fold larger compared to the trypanosomal IAG-NHs, making it more similar to IU-NHs [46]. The crystal structure of the enzyme indeed showed a group-I fold, and surprisingly, neither Trp nor His residue preceding the last Ca^2+^-coordinating Asp were poised for leaving-group activation [73]. The His residue in the β3-α3 loop was suggested as the likely proton donor in the enzymatic reaction. The CU-NH isozyme from the same organism [47] displayed the group I structure expected based on the substrate specificity and also lacked a His241 homolog, which means it takes advantage of an alternative mechanism for leaving-group activation. The structure also revealed a striking, unprecedented peculiarity, that is, the substitution of the active site Ca^2+^ ion with a Na^+^ [73]. This difference is likely due to a unique Glu residue in the N-terminal fingerprint sequence (DCDTAEDD) that interacts with the β3-α3 loop His residue and causes rearrangements in the active site residues that likely favor the binding of the alkali metal ion. This finding also suggests that the cation in the NH active sites plays a role in substrate binding, but has only a minor effect on the polarization of the catalytic water.

### 2.4. NH Quaternary Structure

The oligomerization of NHs to form stable quaternary structures is a well-known phenomenon. However, the inter-monomer interactions are very different in the structural groups, involving distinct secondary structural elements, and have different implications for the enzymatic activity. Here, I analyze the existing data on the mechanism of oligomerization in NHs, along with the structural and functional consequences of quaternary structure formation.

#### 2.4.1. NH Structural Group I: From Monomers to Tetramers in Dynamic Equilibrium?

The structural group I NHs include enzymes that are either non-specific or display a marked preference for pyrimidine nucleoside substrates. The *S. cerevisiae* uridine hydrolase URH1 was reported to elute as a monomer from size-exclusion chromatography [63], while the *C. fasciculata* IU-NH displayed elution volumes characteristic of a tetramer [24]. The crystal structure of the unliganded *Crithidia* IU-NH showed a dimer in the asymmetric unit, but a tetrameric assembly could be generated by the twofold symmetry axis in the orthorhombic crystal [32]. The conserved tetrameric assembly as an asymmetric unique object was confirmed in the structure of the same enzyme in complex with an inhibitor crystallized in a different crystal form [32]. The *L. major* non-specific NH crystallized in a tetrameric arrangement that paralleled what was observed in the *Crithidia* isozyme [74], and this was confirmed by the following structures of several bacterial and archaeal enzymes [33,34,73].

So far, the only report of a monomeric active NH is represented by the NH URH1 purified from baker’s yeast [62,63]. This enzyme was initially characterized for its activity on pyrimidine nucleosides, revealing a marked specificity toward the uridine substrate. More recently, URH1 was shown to be 100-fold more efficient in the hydrolysis of nicotinamide riboside [3]. This activity is crucial for the activation of sirtuins and contributes to the extension of the lifespan of yeast cells. The crystal structure of URH1 has not been reported so far, but an AlphaFold2 model with a high model confidence is available for analysis of the interaction surfaces. Pending experimental validation, this model may prove extremely useful in designing mutations in group I NHs with potentially therapeutic activities to avoid oligomerization.

**Figure 5:**
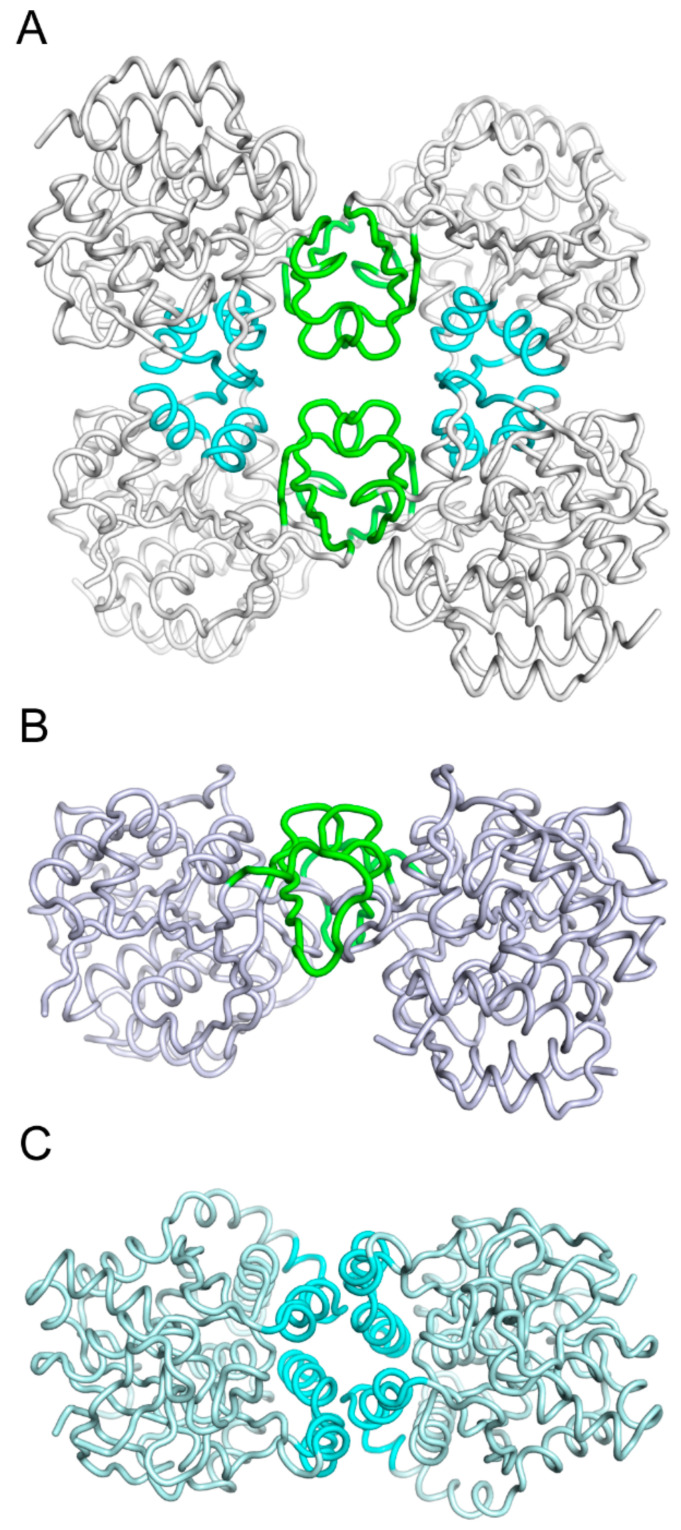
Oligomerization of NH proteins. (**A**) Ribbon diagram of the tetrameric group I IU-NH from *C. fasciculata*. The quaternary structure is achieved through two interaction surfaces, a major (colored green) one largely involving the β8 and β9 strands that are not part of the central core sheet, and a minor (cyan) one that is composed of residues from the loop connecting strand β3 to helix α3, and helices α4.1 and α5.2. (**B**) Ribbon diagram of the dimeric plant NH. This dimer, oriented in the figure as the *C. fasciculata* isozyme in the top panel, is stabilized by extensive interactions between residues corresponding to the major surface in the group I tetramers. The conformation of the secondary structural elements corresponding to the minor interface is conserved; thus, the different amino acid compositions at the β3-α3 junction and α4.1 and α5.2 helices prevent the formation of the tetrameric structure. (**C**) Dimeric group II NH. Deletions in the β8-β9 region and the presence of two additional helices α3.1 and α3.2 in the region joining strand β3 to β4 allow a different mode of intermolecular stabilization.

The group I NH tetramers display a 222 (D2)-point group symmetry, with four perpendicular twofold axes relating the four identical protomers, assembled through two distinct oligomerization surfaces. The major interaction surface spans 920 Å^2^ and involves the two short β-strands outside the main core β-sheet (Figure 5) and connecting loops from two monomers. The nature of the surface is quite diverse in different group I members, with hydrophilic, hydrophobic, and charged residues contributing to the monomer-monomer interaction. High-resolution structures showed that several ordered water molecules are trapped between the protomers and mediate hydrogen bonds that stabilize the monomer-monomer contacts. Comparison of the sequences of several group I NHs in this region reveals a marked diversity in the amino acid composition, surprising in the light of the conserved quaternary arrangement. The second interaction surface is more variable in size, ranging from 674 Å^2^ in the *S. solfataricus* CU-NH to 807 Å^2^ in the RihB/YeiK CU-NH from *E. coli*. This surface is composed of two short helical segments, α4.1 and α5.2, and the initial portion of the active-site loop following strand β3. Analysis of this surface using PISA shows that the interaction is generally computed as weaker, albeit with a wide range of energies, and more likely to dissociate in solution compared to the major interface (Table S3). Thus, it can be envisioned that group I NHs in the cell, under dilute conditions, may exist as dimers as seen for the *S. solfataricus* IAGNH [46]. Nevertheless, it is noteworthy that all group I NHs from bacteria and protozoa and group III NHs from protozoa and metazoa so far characterized crystals as structurally homologous homotetramers, taking advantage of the same minor surface to form the dimer of dimers and with similar overall geometries. It remains to be seen whether this potentially dynamic dimer-tetramer equilibrium bears some relevance with the biological role of NHs. The tetrameric structure may, for instance, favor the formation of transient supermolecular complexes with other nucleobase-processing enzymes such as PRTases.

All plant NHs that have been so far characterized by X-ray crystallography display a homodimeric quaternary structure (Figure 5). Indeed, the enzymes from *P. patens* and *Zea mays* crystallize only as homodimers formed through the major interaction surface [58]. A longer loop region surrounding the antiparallel β-strands involved in dimerization demonstrates extensive interactions and likely has a stabilizing effect. An interesting study on the two plant NHs NSH1 and NSH2 from *A. thaliana*, who cooperate in uridine and xanthosine catabolism [57], produced exciting insights into the effect of protein oligomerization on the enzymatic activity. Both gene products share the NH fingerprint sequence and characteristics of group I NHs, and NSH1 is a uridine hydrolase, the mutation of which leads to accumulation of both uridine and xanthosine, while NHS2 mutants have reduced hydrolytic activity toward inosine and xanthosine. Co-expression in tobacco leaves of the two gene products and inactive mutants elegantly demonstrated that they form heterodimers, and that the interaction with NHS1 activates NHS2, which is otherwise inactive, to engage in xanthosine-inosine NH activity. NHS1-NHS2 heterodimerization enhances the uridine hydrolase activity of NHS1 compared to the homodimers, thus providing the first clear evidence of a long-range effect of different interactions on the major surface toward the active sites. No structural analysis is yet available for these heterodimers, but should undoubtedly provide important information on how oligomerization can affect the arrangement of the amino acids at the active site that are ~25 Å away from the major protomer-protomer interaction surface. Thus, the formation of at least stable dimers is instrumental for maintaining a catalytically competent active site architecture in the *Arabidopsis* group I NHs, and the active site geometry can be likely modulated by different oligomerization states and/or interacting partners. Interestingly, no such hetero-oligomers have been observed in *E. coli*, where the three NHs RihA, RihB, and RihC form homo-oligomers. At least in RihA and RihB, the different natures of the residues involved in the oligomerization on the major interacting surface prevent the formation of heterocomplexes between the different gene products [33,75].

#### 2.4.2. Group II NHs: Stable Dimers with Inter-Subunit Cooperativity

The three IAG-NH isozymes from trypanosomatidae (*T. vivax*, *T. b. brucei*) and bacteria (*Bacillus anthracis*) so far deposited with the Protein Data Bank are homodimers in the crystal structures. The monomer-monomer interface in the unliganded *T. b. brucei* NH spans 960 Å^2^ and involves residues from the two additional helices (α3.1 and α3.2), between strand β3 and helix α3.3, which are not present in the group I enzymes. These helical segments interact with helix α7 and 8 of the opposing monomer (Figure 5). This interaction surface also has a mixed character, as shown for the group I NH dimerization interface, with contributions from both hydrophilic and hydrophobic residues (Table S4). When this IAG-NH is bound to a competitive inhibitor, the folding of the region spanning amino acids 244 to 256 into a helical segment to bring residues in contact with the ligand increases the monomer-monomer interaction surface to 1300 Å^2^.

The importance of the intermolecular contacts in determining the enzymatic activity is confirmed by distinct experiments. First, the *T. vivax* IAG-NH displays half-of-the-sites reactivity, suggesting that a mutual regulation of the two monomers, mediated by the inter-subunit contacts, takes place during catalysis [76]. It is tempting to speculate that binding of a substrate to one protomer induces asymmetry in the dimer, resulting in only one competent active site in each catalytic cycle, as seen, for instance, for the *Pyrococcus furiosus* methionine adenosyl transferase [77]. No structure of half-occupied IAG-NH dimers has so far been reported, and thus the atomic details regulating the mechanism underlying this characterizing kinetic feature are still unresolved. Secondly, the IAG-NH from *T. b. brucei* is allosterically inhibited with a noncompetitive mechanism by divalent cations such as Cu^2+^ and Ni^2+^, binding at the dimer interface via two symmetric His residues in the catalytic loop and preventing its closure to the active conformation [31].

#### 2.4.3. Monomeric Inactive NH-Like Proteins

**Figure 6:**
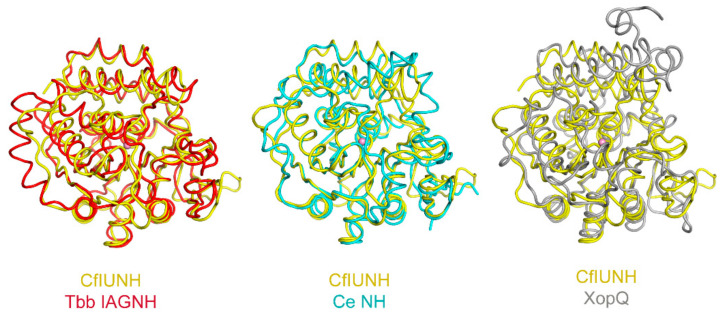
Structural comparison between NH proteins. Pairwise superposition of the *T. brucei brucei* IAG-NH (red), *C. elegans* NH (cyan), and *X. oryzae* XopQ onto the *C. fasciculata* IU-NH (yellow) highlights the conservation of the core b-sheet, while the surrounding secondary structural elements assume diverse orientations, affecting their activity and oligomerization.

The *Xanthomonas* XopQ protein structure provided for the first time a glimpse of an isolated monomer with an NH fold [50]. A computed 2.4-root mean square distance for 246 Cα atoms when XopQ is superimposed onto the *C. fasciculata* IU-NH monomer suggests an overall structural similarity, but with a significant divergence in the relative placement of the secondary structural elements (Figure 6). The monomeric state of XopQ can be largely ascribed to the absence of both the antiparallel β strands contributing to the major interaction surface in group I NHs, which are substituted with a shorter, more direct connecting peptide eight residues long (that not only prevents the formation of contact promoting intermolecular association but also imposes a different conformation in the neighboring structural elements β5 and α9). The regions involved in dimerization in group II NHs are less protruding from the monomer core and are not as exposed as in the *T. vivax* and *T. b. brucei* enzymes, and at least in the crystals so far obtained, are not involved in oligomerization. Although a fine structural comparison between XopQ and group I NHs should consider the limited homology between the amino acid sequences, the absence of the inter-subunit contacts in the monomeric XopQ may also play a factor in maintaining the structural integrity of the catalytic center.

In the *S. degradans* Sde_0182 protein, the C-terminal domain adopts an NH-like fold with a preserved Ca^2+^ ion and an additional β-sandwich domain that interacts with the region corresponding to the dimerization interface. Thus, the presence of an additional domain provides structural stabilization but prevents oligomerization of the NH domain of Sde_0182 [53]. This protein is also catalytically inactive while retaining the ribofuranose-binding capability, and further reinforces the concept that the assembly of dimers or tetramers in NHs is a primary means to achieve a catalytically competent active site to achieve N-glycosidic bond hydrolysis.

#### 2.4.4. Beyond Quaternary Structure: Protein-Protein Interactions Involving NHs

The interaction of NHs with other proteins has been explored only to a limited extent. The quaternary structure of NHs that may be instrumental in the formation of transient multienzyme complexes to facilitate substrate funneling between active sites has been proposed. The *S. cerevisiae* URH1 provides a convenient model system for the study of the NH interactome, as genetic manipulation of yeast can be conveniently used to this end. Using the two-hybrid approach, the IMPACT homolog protein YIH1 is identified as a URH1 interactor [78]. YIH1 competes with the Gcn2p eIF2 kinase to bind to Gcn1, and presumably regulates the translation under starvation conditions. Instead, a comprehensive affinity capture followed by mass-spectrometry analysis [79] revealed interactions with several different polypeptides, including physical association with BLOC-1 complex subunit BLI1 involved in endosomal maturation, mitochondrial transcription factor 2, and proteasome subunit α type-1. RNA capture analysis links URH1 to the ribosome-associated ATPase chaperone SSB2 [80], and the yeast NH has also been co-purified with Hsp70-SSB [81]. In *E. coli*, several different approaches have been used to detect protein-protein interactions to define a whole cell interactome. When using histidine-tagged RihB as bait, the large ribosomal subunit UL4, ribosomal alanine N-acetyltransferase, NH_3_-dependent NAD synthetase and dTTP/UTP pyrophosphorylase were copurified [82]. Moreover, RihB was eluted when the uroporphyrinogen decarboxylase HemE was used as bait, together with an uncharacterized oxidoreductase YhhX and the phosphoribosylamine-glycine ligase that participates in purine nucleotide synthesis.

Genetic interaction screens may better identify pathways in which NHs may act. Using combinations of digenic mutants [83], the *E. coli* RihB was shown to affect the growth of cells when either *cspD* (encoding for a cold shock protein), *rsgA* (ribosome small subunit-dependent GTPase), or *yfbJ* (inner membrane protein) were simultaneously inactivated. The RihA protein, despite its similar enzymatic activity and substrate specificity, was associated with 16 genes encoding for proteins with very diverse functions. Given the diverse roles of the possible physical interactors, it would be speculative to draw definitive conclusions on a shared physiological role other than nucleoside metabolism. Molecular validation of the high-throughput data is required to ascertain the association of NH enzymes with specific metabolic networks.

### 2.5. NH Structures and Drug Design

#### 2.5.1. Antiprotozoan Compounds Targeting Purine-Specific NHs

As stated previously, mammals take advantage of NP activity to detach nucleobases from nucleosides. The free nitrogenous bases can ultimately be subjected to either oxidation to uric acid for purines or reduction to β-alanine/β-aminoisobutyrate for pyrimidines, or recycled to mononucleotides through condensation with PRPP via PRTase-catalyzed reactions. Moreover, all mammalian genomes encode for enzymes that catalyze the de novo synthesis of the nucleobase rings, starting from low-molecular-weight compounds. In sharp contrast, trypanosomes are purine-auxotrophic organisms since they lack all enzymatic activities required for purine nucleotide synthesis [84]. Thus, these protozoa rely on salvage pathways for uptake from the host of these RNA and DNA components. It has been shown that more than 85% of the nucleosides taken up in *C. fasciculata* undergo N-glycosidic bond cleavage [22]. NP activity or NP-encoding genes are not present in trypanosomes that hence take advantage of the NH-catalyzed N-glycosidic bond cleavage for purine base salvage [84]. These metabolic differences between mammalian hosts and trypanosomes make the purine salvage pathway of the parasites an attractive target for drug design.

**Figure 7:**
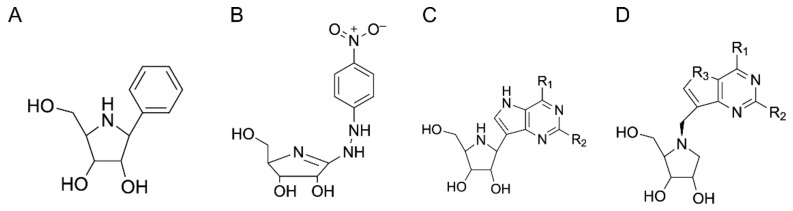
Iminoribitol-based NH inhibitors. (**A**) Substituted phenyliminoribitols are effective, transition state-like inhibitors of the *Crithidia* IU-NH. (**B**) 4-nitrophenyl riboamidrazone, a nanomolar inhibitor of the *C. fasciculata* isozyme. (**C**) Immucillins, originally identified as transition state-like NP inhibitors, are also effective inhibitors of the group II trypanosomal IAG-NHs and IG-NH. (**D**) Methylaryl iminoribitols show high affinity toward trypanosomal IAG-NHs with high selectivity, being much weaker NP inhibitors.

The first NH inhibitors were designed based on the transition state structure of the enzymatic reaction, as determined by multiple kinetic isotope effects [27]. The partial double-bond character of the C1’-O4’ linkage and the partial positive charge developing in the furanose ring were best mimicked by a (2*R*, 3*R*, 4*S*)-2-hydroxymethyl-3,4-pyrrolidinediol (iminoribitol) moiety (Figure 7) [85]. A non-scissile carbon-carbon linkage was used to link aromatic moieties at the C1’ carbon of iminoribitol. The IU-NH from *C. fasciculata* was inhibited by phenyl-iminoribitols with nanomolar inhibition constants. Iminoribitols linked to purine rings (immucillins) were less effective inhibitors of this isozyme, but displayed nanomolar dissociation constants toward the group II purine-specific IAG-NH and IG-NH isozymes [86]. These compounds, however, are also potent inhibitors of the human NPs and are thus not amenable to therapeutic use. It should be noted that although humans have alternative pathways for purine base synthesis or salvage, the lack of NP activity leads to impairment of T cell proliferation and ultimately a form of immunodeficiency (PNP-SCID) [11]. Indeed, immucillins have undergone clinical trials for the treatment of T cell-mediated diseases such as leukemias, lymphomas, and psoriasis (https://www.clinicaltrials.gov/ct2/results?term=Forodesine) (accessed on 24 November 2021). Recently, immucillin-A (a 9-deazaadenine linked via a C-glycosidic bond to the iminoribitol C1’ atom, also known as BCX4430 or Galidesivir) was demonstrated to be effective against filovirus infection, where it is converted to the corresponding triphosphate to inhibit viral RNA polymerase transcriptional activity [87].

Alternatives to immucillin-like structures showing strong selectivity toward protozoan NHs have been pursued through structure-based drug design approaches. A series of N-arylmethyl compounds (Figure 7) were synthesized based on the iminoribitol scaffold but with a linkage between the base-mimicking moiety and the Ca^2+^-interacting portion mediated by a methylene bridge to the N4’ atom [88,89,90]. Such compounds displayed 4–360 nM inhibition constants of trypanosomal IAG-NHs with at least 1000 selectivity in inhibition of the NHs over human PNP [91]. Albeit requiring a higher concentration compared to pentamidine or diminazene aceturate, the UAMC-00363 compound was effective as a trypanocidal in culture. The compound also showed effective clearance of the parasites in an animal infection model on treatment for five days at a dose of 50 mg/kg. These results showed for the first time that inhibitors specific to the trypanosomal IAG-NHs can indeed act as trypanocidal compounds. The bioavailability of the compounds, namely the transport by equilibrative nucleoside transporters across the protozoan membrane [8,91], requires further optimization to provide a panel of compounds that can be further tested in in vivo models of infection.

The crystal structures of trypanosomal IAG-NHs provided several clues as to the inhibitor determinants that are responsible for the high affinity. The optimal coordination of the active site Ca^2+^ ion by the ribose-like moiety of the active site ligands is crucial. Indeed, a comparison of the high-resolution structures of the IAG-NH from *T. b. brucei* bound to Immucillin-H (0.7 nM K_i_) and the UAMC-00363 showed that the only appreciable difference in enzyme-inhibitor contacts is the five-membered ring [31]. The structures of N-arylmethyl iminoribitols differing at the aromatic portion helped define which protein residues can be exploited for hydrogen-bonding interactions to improve affinity. It is noteworthy how limited differences at the active sites of the *T. b. brucei* and *T. vivax* IAG-NHs may result in an up to 10-fold difference in K_i_ values, thus underscoring the need to validate each inhibitor panel with the actual target from the pathogen.

The *T. b. brucei* IAG-NH was also inhibited by metalorganic compounds, such as a Ni^2+^·Tris complex formed at a basic pH [31]. The Ni^2+^ is coordinated by the Tris molecule and ordered solvent, and only residue Asp14 of the enzyme directly interacts with the ion. The hydroxyls of the Tris molecule partly mimic the ribose O3’ and O5’ groups, underscoring the importance of these subsites for active-site ligation. It is noteworthy that the transition metal ion can closely approach (3.8 Å) the active site Ca^2+^, implying that the alkali earth ion charge is substantially shielded by the coordination of the active site aspartates. Although yet unexploited, these findings clearly show that alternatives to ribosyl-containing compounds could be engineered as IAG-NH inhibitors to overcome issues of bioavailability and cross-reactivity with mammalian NPs. Crystallographic fragment-screening approaches may provide further clues as to the process of antiprotozoan drug design.

Another approach to enzymatic inhibition could be to target regions other than the active site by changing the equilibrium between active and inactive conformations, favoring the latter through non-competitive inhibition. IAG-NH is an enzyme with half of the sites’ reactivity and requires cooperation of both subunits for efficient catalysis. Divalent transition metal ions such as Cu^2+^, Mn^2+^, Co^2+^, Zn^2+^, and Ni^2+^ are non-competitive inhibitors of the enzyme, Cu^2+^ ions being the most potent with K_ii_ = 0.9 µM. The cocrystal structure of IAG-NH with Ni^2+^ ions revealed that the ion binds at the monomer-monomer interface, interacting with two histidine residues from opposing subunits. The coordinated ion locks the catalytic loops in a semi-open conformation, preventing the closure of the active-site cavity and the positioning of key catalytic residues [31]. Thus, the stabilization of an inactive conformation of IAG-NH by promoting specific interactions mediated by the quaternary assembly of the protein may represent a tempting alternative for the development of lead compounds that depart from the substrate-like compounds containing ribosyl- or iminoribitol-like moieties.

Although NHs are extremely attractive drug targets due to their absence in mammalian hosts, it remains to be seen whether these enzymes may play a crucial role in the metabolic pathways of other pathogens. Pathogens such as *T. vaginalis* are both purine and pyrimidine auxotrophic, and the dependence of their life cycle on the genome-encoded NHs could be inferred [92,93,94]. The finding that the yeast NH URH1 is active on pyridine nucleosides (such as nicotinamide riboside) links NHs to components of the redox cofactors NAD^+^ and NADPH, and further suggests that the enzyme may have a specialized—yet, so far not fully identified—role in different organisms [14]. Indeed, the substrate specificity/preference of NHs may be modulated through substitutions in several amino acids, as demonstrated for the *E. coli* YeiK/RihB but also from the differences in the substrate preference of isozymes within the same organism.

#### 2.5.2. NHs in Antiprotozoan Vaccination Strategy

As is the case for other protozoan parasites, members of the *Leishmania* genus also encode for two NHs in their genome, and their expression has been demonstrated in several isolates of *L. infantum* and *L. donovanii* [74,95,96]. These isozymes can be inhibited by several immucillins, most efficiently by Immucillin-A, to impair *Leishmania* mastigotes’ replication in vitro and in vivo. Moreover, the isozyme NH36 of *L. donovanii*, the causative agent of visceral leishmaniasis, was found to be one of the most immunogenic proteins from mastigote extracts, and the recombinant NH36 protein (part of the Leishmune^©^ vaccine) is effective at eliciting a curative and protective immune response against murine and canine diseases. Three NH36 subsequences (1-103, 104-198, 199-314) were used to define minimal epitopes retaining adequate immunogenic properties. Interestingly, the C-terminal (termed F3) region expressed in *E. coli* co-administrated with saponin elicited a ~36% increase in the protective response against *L. chagasi*, mostly mediated by IFN-γ/IL-10 CD4^+^ T cells [97]. Although no details on the folding of the fragment expressed are yet available, the F3 region contains the β-strands that mediate protein oligomerization. It remains to be assessed whether and how the oligomerization state of the recombinant vaccine influences the immune response.

#### 2.5.3. NHs in Gene-Directed Enzyme-Prodrug Activation Therapy

Gene-directed enzyme prodrug activation therapy (GDEPT) is a therapeutic approach relying on the enzymatic conversion of a non-toxic prodrug to its active form by the product of an exogenous gene expressed in the target cells, typically of neoplastic nature. Severely limited by the targeting of the gene to the tumor cells and the transduction efficiency, GDEPT can greatly benefit from the bystander effect where dying cells transfer the active compound via either passive diffusion or through tight junctions.

**Figure 8:**
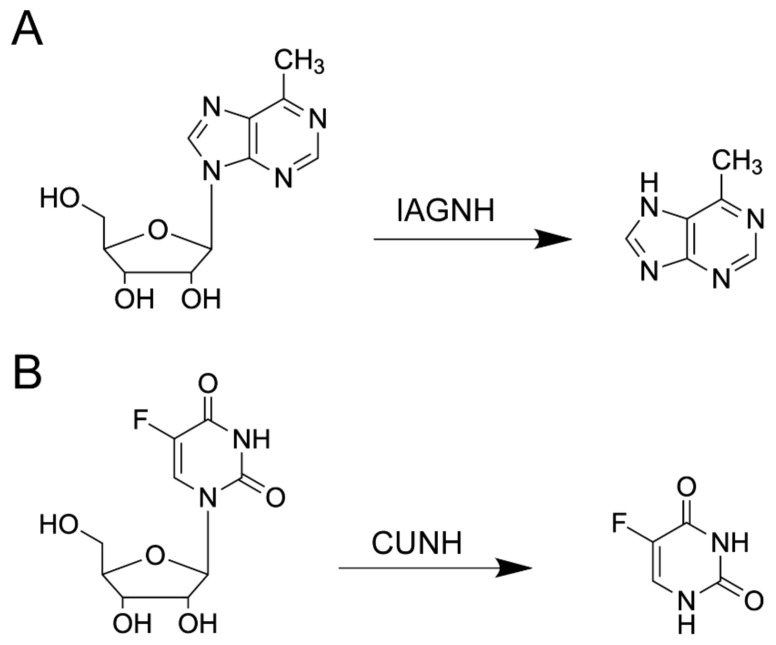
Prodrug activation by NHs. (**A**) IAG-NHs efficiently convert the prodrug 6-methylpurine riboside to 6-methylpurine, whose incorporation in RNA affects protein synthesis. (**B**) Non-specific IU-NHs or pyrimidine-selective CU-NHs hydrolyze the N-glycosidic bond in 5-fluorouridine, leading to release of 5-fluorouracil. The fluorinated pyrimidine is incorporated in nucleotides that hinder the RNA secondary structure, and the deoxy mononucleotide inhibits the key enzyme thymidylate synthase.

Although NHs have clearly evolved to catalyze reactions on naturally-occurring nucleoside substrates (Figure 8), their active site characteristics allow for the cleavage of the N-glycosidic bond in non-natural derivatives. Many pyrimidine nucleosides substituted at the C5 position of the nitrogenous base have been used to probe substrate specificity and catalytic mechanisms in several NHs.

The IAG-NH enzyme from *T. vivax* is active on nucleoside substrates such as 6-methylpurine riboside, the hydrolytic product of which, 6-methylpurine, is toxic as it interferes with protein and RNA synthesis, as well as adenine salvage. An innovative liposome-based delivery system has been developed, where the IAG-NH enzyme is encapsulated in liposomes with the bacterial OmpF transporter embedded [98]. The enzyme is segregated in the vesicular structure, and the OmpF protein allows for the entry of the nucleoside prodrug and efflux of the 6-methylpurine bioactive toxic compound. An alternative reactor was also obtained using amphiphilic triblock copolymers [99]. In both cases, the enzyme retained activity, albeit with a reduced efficiency that was associated with a lower prodrug concentration within the reactor. These delivery systems represent an attractive alternative to gene-based delivery systems, and their application in cancer therapy warrants further investigation.

The pyrimidine-preferring RihB/YeiK NH from *E. coli* has a high catalytic efficiency toward 5-fluoruridine [33], and the product of the hydrolytic reaction is 5-fluorouracil (5-FU), one of the first chemotherapeutic agents used against solid tumors. Cellular enzymes convert 5-FU to the corresponding nucleotide and deoxynucleotide. The fluorinated nucleotide is incorporated into RNA molecules, leading to faulty ribosomal assembly. The enzyme thymidylate synthase is inhibited by 5-FdUMP, depriving cells of thymidine and leading to apoptosis. A strong bystander effect makes this approach extremely efficient to reduce residual disease. However, 5-FU is detoxified in the liver through the action of the dihydropyrimidine dehydrogenase enzyme, limiting its availability for condensation with PRPP to form the corresponding mononucleotide. At the present, 5-FU derivatives such as capecitabine are preferred for use in patients due to their bioavailability and efficacy. The RihB enzyme has been proposed as a possible tool for GDEPT against solid tumors, taking advantage of its catalytic efficiency toward 5-fluorouridine, which is more bioavailable and induces fewer side effects than 5-FU [33]. Two site-specific mutants of RihB with enhanced rates of 5-FU liberation have also been characterized. When delivered to the tumor, an appropriate NH enzyme would be expected to allow faster accumulation of 5-FU and its activation by PRTases, thus enhancing the antitumoral activity. Such results were indeed obtained when the *E. coli* PNP was delivered using attenuated *Salmonella typhimurium* to cancer cells to activate 6-methylpurine 2’-deoxyriboside [100,101]. Hence, the potential use of NHs in cancer-directed GDEPT must be adequately explored since their activity on bioavailable nucleoside analogs may reveal a superior capability for the concentration of toxic compounds in neoplastic cells.

The quaternary structure of NHs may prove extremely relevant in the design of prodrug activators. Indeed, the construction of chimeric enzymes catalyzing subsequent reactions required for activation of the toxic drug and thus improved efficacy has been tested in several cases. The product of the hydrolytic reaction catalyzed by NHs is not toxic to cells per se, but rather requires further condensation with PRPP through the action of PRTases, to yield the mononucleotide that will eventually either be incorporated in RNA molecules, or after reduction to the corresponding nucleoside, will interfere with thymidine synthesis or DNA replication. Thus, a chimeric NH-PRTase enzyme would encode for the two crucial activities that yield a mononucleotide, for instance, 5-FUMP, which cannot escape the cell and will lead to antitumoral cytotoxicity. PRTases are known to oligomerize to quaternary structures that range from dimers to tetramers [102,103], and their fusion with oligomeric NHs may lead to the formation of large aggregates that may display suboptimal activity if 5-FU is activated. Thus, application of engineering techniques aimed at preventing NH oligomerization while retaining the crucial enzymatic activity is a field that offers opportunities for the development of NH-based prodrug activators to be tested in disease models.

## 3. Conclusions

Initially considered redundant or exclusive to nucleobase-auxotrophic parasites, NHs are now recognized as widespread and possessing extremely diversified functions in different organisms. The field will largely benefit from close-knit interaction between structural studies, molecular analysis of mutants, and validation of high-throughput genetic analysis to ascertain the role of the enzymes and related proteins in each organism. The catalytic versatility of NHs warrants further exploration of their therapeutic potential as prodrug activators, and they remain attractive targets for development of drugs against parasitic diseases.

## Supplementary Materials

The following are available online at https://www.mdpi.com/article/10.3390/ijms23052576/s1.
