# Supplementary material for: Structure, Oligomerization and Activity Modulation in N-Ribohydrolases
Source: Int J Mol Sci. 2022 Feb 25;23(5):2576. doi: 10.3390/ijms23052576 (PMC8910321; doi:10.3390/ijms23052576)
Supplement: Supplementary file 1 [file ijms-23-02576-s001.zip › ijms-1566228-supplementary.pdf]

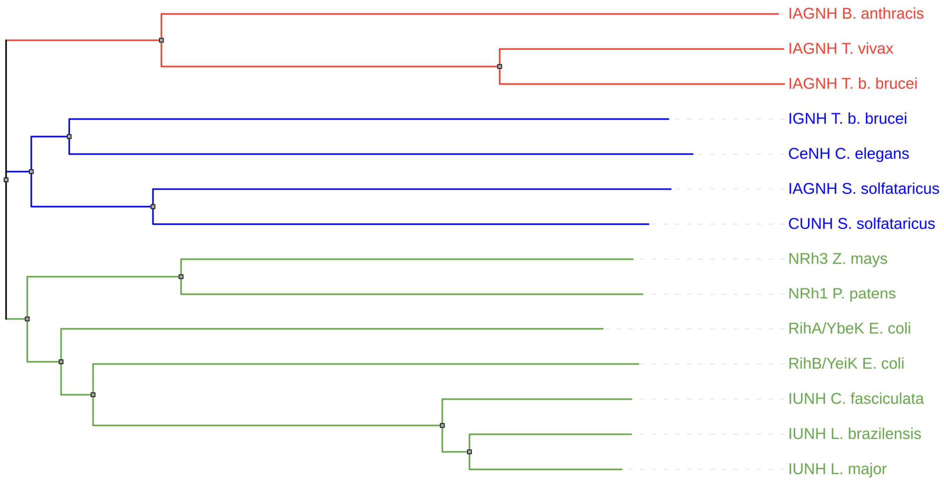

Supplementary Figure S1. Evolutionary relationships between NHs. The amino acid sequences of NHs whose crystal structures have been determined were aligned using ClustalOmega. The phylogenetic tree shows how NHs belonging to different structural groups (group I in green, group II in red, and group III and non-canonical in blue) cluster in different branches.

Table S1. Structural differences between NH proteins.

Protein.	PDB Code	Cf IU-NH (group I)	Tbb IAG-NH (group II)	Ce NH (group III)	XopQ1 (inactive)
**Cf IU-NH (group I)**	2MAS	-	1.37	1.08	2.98
**Tbb IAG-NH (group II)**	4I71	180	-	1.68	2.74
**Ce NH (group III)**	5MJ7	202	200	-	3.50
**XopQ1 (inactive)**	4P5F	206	176	209	-

The root mean square distance (Å) between pairs of proteins is reported in above the diagonal, and the number of corresponding number of Cα atoms used in the calculation is reported below the diagonal. Superposition was performed in Pymol (http://www.pymol.org (accessed on 24 November 2021)) using the *super* command. Cf, *Crithidia fasciculata*; Tbb, *Trypanosoma brucei brucei*; Ce, *Caenorhabditis elegans*; Xo, *Xanthomonas oryzae*.

Table S2. Structural similarity between group I NHs.

	Cf IU-NH	Lm IU-NH	Lb IU-NH	Ec RihA	Ec RihB	Ss CU-NH	Pp NRh1	Zm NRh3
**Cf IU-NH**	-	0.29	0.37	0.78	0.62	1.18	1.05	0.98
**Lm IU-NH**	252	-	0.33	0.82	0.61	1.36	0.93	1.03
**Lb IU-NH**	277	247	-	0.79	0.58	1.18	0.98	0.84
**Ec RihA**	243	228	238	-	0.84	1.01	1.18	0.87
**Ec RihB**	242	218	239	259	-	1.20	1.24	1.17
**Ss CU-NH**	208	210	211	210	207	-	1.04	0.93
**Pp NRh1**	244	203	222	233	240	209	-	0.54
**Zm NRh3**	246	216	213	217	245	215	259	-

Above the diagonal, the root mean square distance (Å) between the Cα atoms for the corresponding pair of proteins after superposition. The number of Cα atoms used in the calculation is reported below the diagonal. Superposition was performed in Pymol (http://www.pymol.org (accessed on 24 November 2021)) using the *super* command. Cf, *Crithidia fasciculata*; Lm, *Leishmania major*; Lb, *Leishmania braziliensis*; Ec, *Escherichia coli;* Ss, *Sulfolobus solfataricus*; Pp, *Physcometrella patens*; Zm, *Zea mays*. Here, the Ss CU-NH is included to highlight its structural divergence, despite sharing the tetrameric quaternary structure and enzymatic parameters.

Table S3. Interaction surfaces in dimeric and tetrameric (group I and III) NHs.

Organism, Protein	PDB	Resolution (Å)	Major Surface (Å^2)	N_{HB}	N_{SB}	N_{SS}	ΔG_b. kcal/mol	Minor Surface (Å^2)	N_{HB}	N_{SB}	N_{SS}	ΔG_b, kcal/mol
***C. fasciculata,* IU-NH**												
unliganded	1MAS	2.5	875	19	2	0	−15.7	755	4	0	0	−11.6
+ inhibitor	2MAS	2.3	869	23	0	0	−18.6	801	2	0	0	−12.3
***L. major,* IU-NH**	1EZR	2.5	930	16	0	0	−14.4	791	4	2	0	−13.2
***L. braziliensis,* IU-NH**	5TSQ	1.5	1100	15	0	1	−27.3	847	6	0	0	−17.5
***E. coli,* RihA/YbeK CU-NH**												
+ribose	1YOE	1.8	1248	15	0	0	−27.7	865	6	6	0	−14.7
+inhibitor	3G5I	2.1	1255	13	0	0	−26.8	807	4	7	0	−14.3
***E. coli,* RihB/YeiK CU-NH**												
unliganded	3MKM	2.2	869	4	0	0	−14.6	854	7	0	0	−14.3
+glycerol	1Q8F	1.7	916	2	0	0	−15.0	825	8	0	0	−15.3
+inhibitor	3MKN	2.0	851	10	0	0	−16.2	805	2	0	0	−12.9
+inosine	3B9X	2.3	912	2	0	0	−14.2	861	7	0	0	−14.2
***S. solfataricus,* CU-NH**	3T8J	1.6	1407	20	0	0	−21.6	670	2	0	0	−10.0
***S. solfataricus,* IAG-NH**	3T8I	1.8	1191	10	4	0	−14.6	706	0	4	1	−18.2
***P. patens,* PpNRh1**	4KPN	3.4	1375	11	1	0	−22.4	-	-	-	-	-
***Z. mays,* ZmNRh3**	4KPO	2.5	1290	10	0	0	−23.7	-	-	-	-	-
***C. elegans,* NH**	5MJ7	1.7	981	17	2	0	−12.6	891	12	2	0	−10.3
***T. brucei brucei,* IG-NH**	3FZ0	2.5	1007	12	0	0	−17.1	787	7	0	0	−11.4

Analysis of the oligomerization interfaces in group I (dimeric and tetrameric) and group III (tetrameric) NHs. Surfaces, number of hydrogen bonds (N_{HB}), salt bridges (N_{SB}), number of disulfide bonds (N_{SS}), and binding ΔG (ΔG_b) were computed using PISA (https://www.ebi.ac.uk/pdbe/pisa/ (accessed on 24 November 2021)).

Table S4. Interaction surfaces in the dimeric group II NHs.

Organism, Protein	PDB	Resolution (Å)	Buried Surface (Å^2)	N_{HB}	N_{SB}	N_{SS}	ΔG_b, kcal/mol
***T. vivax*, IAG-NH**	1HOZ	1.6	890	4	0	0	−14.6
+ *inhibitor*	2FF2	2.2	1264	8	0	0	−27.5
***T. brucei brucei*, IAG-NH**	4I70	1.6	959	8	4	0	−13.0
+ *inhibitor*	4I71	1.3	1300	14	4	0	−24.6
***B. anthracis* NH**	2C40	2.2	951	0	4	0	−15.3

Analysis of the oligomerization interfaces in group II NHs. The *B. anthracis* structure has been released, but no associated publication is reported. Surfaces, number of hydrogen bonds (N_{HB}), salt bridges (N_{SB}), number of disulfide bonds (N_{SS}), and binding ΔG (ΔG_b) were computed using PISA (https://www.ebi.ac.uk/pdbe/pisa/ (accessed on 24 November 2021)).
